# Causal relevance of clonal haematopoiesis with cardiac disease and adverse remodelling: a Mendelian randomisation study

**DOI:** 10.1136/openhrt-2025-003602

**Published:** 2025-10-17

**Authors:** Alec Peter Morley, Maddalena Ardissino, Paul Carter, Betty Raman, Adam J Mead, Pedro M Quiros, George S Vassiliou, Zahra Raisi-Estabragh

**Affiliations:** 1 Gonville and Caius College, University of Cambridge, Cambridge, UK; 2 Department of Medicine, School of Clinical Medicine, University of Cambridge, Cambridge, UK; 3 British Heart Foundation Cardiovascular Epidemiology Unit, Department of Public Health and Primary Care, University of Cambridge, Cambridge, UK; 4 Institute of Medical Sciences, Imperial College London, London, UK; 5 Victor Phillip Dahdaleh Heart and Lung Research Institute, University of Cambridge, Cambridge, UK; 6 Division of Cardiovascular Medicine, Radcliffe Department of Medicine, National Institute for Health Research Oxford Biomedical Research Centre, University of Oxford, Oxford, UK; 7 Medical Research Council (MRC) Weatherall Institute of Molecular Medicine (WIMM) and NIHR Biomedical Research Centre, University of Oxford, Oxford, UK; 8 Department of Biochemistry and Molecular Biology, Instituto Universitario de Oncología (IUOPA), Universidad de Oviedo, Oviedo, Spain; 9 Cambridge Stem Cell Institute, University of Cambridge, Cambridge, UK; 10 Department of Haematology, University of Cambridge, Cambridge, UK; 11 William Harvey Research Institute, NIHR Barts Biomedical Research Centre, Queen Mary University of London, London, UK; 12 Barts Heart Centre, St Bartholomew’s Hospital, Barts Health NHS Trust, London, UK

**Keywords:** Magnetic Resonance Imaging, Genetics, Heart Failure, Risk Factors

## Abstract

**Background:**

Many observational studies highlight clonal haematopoiesis (CH) as a novel determinant of cardiovascular disease (CVD). However, disentangling cause and effect from important confounders, such as age and smoking, is challenging.

**Objectives:**

Mendelian randomisation (MR) was used to assess the causal relationships of CH with (1) major CVD outcomes associated with adverse remodelling, and (2) cardiovascular magnetic resonance (CMR) phenotypes which have not been examined previously.

**Methods:**

Uncorrelated (r^2^<0.001), genome-wide significant (p<5×10^−6^) single nucleotide polymorphisms were extracted from Genome-Wide Association Study summary statistics for CH (any subtype), gene-specific CH subtypes (*DNMT3A* and *TET2*), and CH clonal size subtypes (small clone and large clone). Mendelian Randomisation using a Robust Adjusted Profile Score (MR-RAPS) was used for analyses on outcomes of atrial fibrillation (AF), heart failure and 13 CMR phenotypes. Multiple comparisons in the discovery analyses were accounted for by Benjamini–Hochberg correction.

**Results:**

Both *DNMT3A*-CH and small-clone-CH were associated with increased AF risk. Overall-CH was associated with larger left ventricular end-diastolic volume. *DNMT3A*-CH was associated with larger right atrial size, and left and right ventricular end-diastolic volumes. *TET2*-CH was associated with higher myocardial native T1 time. Small-clone-CH was associated with larger left atrial size and lower aortic distensibility.

**Conclusions:**

Common forms of CH are associated with higher AF risk and adverse remodelling patterns comprising larger atrial and ventricular sizes, myocardial fibrosis, and reduced aortic compliance. Using MR methods, this study triangulates previous observational studies and provides new evidence to support likely causal links between CH and CVD. This study, for the first time, describes associations of CH with adverse CMR phenotypes suggesting early remodelling patterns; these changes may indicate a window of opportunity for intervention such as by risk stratification and early preventative strategies to improve patient outcomes; however, further examination of the utility of such interventions is warranted.

**WHAT IS ALREADY KNOWN ON THIS TOPIC**

- Clonal haematopoiesis (CH) is proposed as a novel marker of biological ageing and adverse cardiovascular risk, however potential confounding (such as ageing and smoking) and reverse causation severely limit causal inference from existing observational research.

**WHAT THIS STUDY ADDS**

- This is the first genetics-based inferential study to demonstrate likely causal associations between CH (of differing driver mutations and clone size) with increased risk of atrial fibrillation and adverse image-derived measures of cardiac structure and function.

**HOW THIS STUDY MIGHT AFFECT RESEARCH, PRACTICE OR POLICY**

- This study indicates a likely causal role of CH as a driver of cardiovascular disease (CVD) and adverse subclinical cardiovascular remodelling. Interventions that may modify these disease trajectories would represent novel targets for disease prevention and merit dedicated research.
- These findings support the potential value of integrating CH sequencing into personalised CVD prevention strategies.
- Future work on pathways to clinical translation is needed to evaluate feasibility and population health benefits.

## Background

 Ageing is associated with the acquisition of somatic mutations. While most have no functional significance, some somatic mutations provide a cell survival advantage leading to establishment and outgrowth of mutation-bearing clones, with potentially deleterious outcomes. Clonal haematopoiesis (CH) refers to the clonal expansion of individual haematopoietic stem cells and their progeny driven by somatic mutations, in the absence of haematological malignancy.^1^ Around 70% of CH cases with known leukaemia-associated mutation drivers occur in the genes encoding epigenetic regulators *DNMT3A* and *TET2*, with most of the remainder driven by mutations in genes for chromatin regulator *ASXL1*, DNA damage response proteins *PPM1D* and *P53*, splicing factors *SF3B1* and *SRSF2*, or the tyrosine kinase *JAK2*.^2^

CH is the precursor of most myeloid neoplasms, but less than 2% of CH carriers develop these cancers with risk varying by mutant gene and clone size.^3^ This modest elevation in myeloid cancer risk explains only a small part of the increased overall mortality risk associated with CH,^4^ much of which has been attributed to augmented cardiovascular disease (CVD) susceptibility. Growing evidence from observational studies demonstrates independent associations of CH with a range of CVD outcomes, including atherosclerotic disease,^5^ atrial fibrillation (AF),^6^ heart failure (HF)^7^,^10^ and aortic aneurysms.^11^ The magnitude of these relationships and CH prevalence is comparable to that of traditional risk factors, heralding CH as a novel CVD determinant. Mechanistic studies have implicated inflammatory mechanisms^12^ related to augmented mutant macrophage inflammation and inflammasome activity. However, others have suggested the associations could be influenced by confounding from major shared risk factors, such as smoking,^13^ accelerated aging,^14^ shared genetic risk or reverse causation.^15^

Mendelian randomisation (MR) is a genetic epidemiological method that leverages the random inheritance of genes to investigate exposure-outcome relationships within the construct of a natural experiment.^16^ This methodology results in random distribution of confounders independent of genetic risk,^17^ similar to a randomised study, greatly mitigating against the influence of biases such as reverse causation and unmeasured confounding. Thus, MR offers the opportunity to support causal associations between CH and CVD outcomes, provided certain assumptions are met.

Cardiovascular magnetic resonance (CMR) provides highly detailed organ-level information about health and disease patterns, indicating preclinical disease states and providing insight into potential underlying disease processes.^18 19^ CMR is the reference modality for evaluation of cardiovascular structure and function, and uniquely provides non-invasive information about myocardial character almost akin to a tissue biopsy.^20^ CMR has huge potential for risk stratification of people with CH; however, relationships of CH with CMR phenotypes have not been previously assessed with genetic or observational studies, representing an important knowledge gap, which this study aims to address.

This study uses MR to evaluate causal associations of different CH subtypes and clone sizes with: (1) CVD outcomes associated with adverse remodelling (AF and HF), and (2) 13 genetically predicted CMR-derived phenotypes of cardiovascular structure and function. We validate our findings across a range of sensitivity and independent replication analyses.

## Methods

This study used publicly available Genome-Wide Association Study (GWAS) summary data available to download at cited sources.^21^,^30^ A summary of sources is provided in table 1.

**Table 1:** GWAS data sources for instrumental variable selection

Phenotype	Study or consortium	Ancestry	Cases/controls	Case definition	Control definition	GWAS units	PMID
Exposures for primary analysis							
Overall-CH	Kar *et al*^21^	EUR	10 203/173 918	Any CH (*ASXL1, ATM, BCOR, BCORL1, BRAF, BRCC3, CALR, CBL, CSF1R, DNMT3A, EZH2, FLT3, GNAS, GNB1, IDH1, IDH2, JAK2, KDM6A, KIT, KRAS, MPL, MYD88, NPM1, NRAS, PHF6, PIGA, PPM1D, PRPF40B, RAD21, RUNX1, SF1, SF3A1, SF3B1, SMC1A, SMC3, SRSF2, STAG2, STAT3, TET2, TP53, U2AF1, U2AF2, ZRSR2* mutations)	No CH	Log(OR)	35 835 912
*DNMT3A*-CH	Kar *et al*^21^	EUR	5185/1 73 918	CH (*DNMT3A* mutation)	No CH	Log(OR)	35 835 912
*TET2*-CH	Kar *et al*^21^	EUR	2042/173 918	CH (*TET2* mutation)	No CH	Log(OR)	35 835 912
Large-clone-CH	Kar *et al*^21^	EUR	4049/173 918	CH (Large-clone: VAF≥0.1)	No CH	Log(OR)	35 835 912
Small-clone-CH	Kar *et al*^21^	EUR	6154/173 918	CH (Small-clone: VAF<0.1)	No CH	Log(OR)	35 835 912
Exposures for replication analysis							
Overall-CH	Kessler *et al*^22^	EUR	25 657/342 869	CH (with or without mosaic chromosomal alteration)	No CH	Log(OR)	36 450 978
*DNMT3A*-CH	Kessler *et al*^22^	EUR	16 219/342 869	CH (*DNMT3A* mutation)	No CH	Log(OR)	36 450 978
*TET2*-CH	Kessler *et al*^22^	EUR	3918/342 869	CH (*TET2* mutation)	No CH	Log(OR)	36 450 978
Exposures for validation analysis							
Systolic blood pressure	Evangelou *et al*^23^	EUR	757 601	N/A	N/A	mmHg	30 224 653
Outcomes							
Atrial fibrillation	Nielsen *et al*^24^	EUR	60 620/970 216	Clinically diagnosed atrial fibrillation or flutter UK Biobank and Trøndelag Health Study cohorts: ICD-9 427.3 ICD-10 I48	No history of atrial fibrillation, flutter or other arrhythmias	Log(OR)	30 061 737
Heart failure	Levin *et al*^25^	EUR	95 524/1270 968	Diagnosis of heart failure by physician, or healthcare record, and corroborated on self-report	No history of heart failure	Log(OR)	36 376 295
Cardiac structure and function	Pirruccello *et al*^26^	EUR	45 504	UK Biobank participants (Ascending aorta diameter; proximal pulmonary artery diameter; LVEDV; LVEF*; RA FAC*; RA Max; RVEF*; RVEDV)	N/A	1-SD	35 697 867
Left atrial maximum volume and left atrial total ejection fraction	Ahlberg *et al*^27^	EUR	35 658	UK Biobank participants (LA Max & LATEF*)	N/A	1-SD	34 338 756
Left ventricular mass	Khurshid *et al*^28^	EUR	43 230	UK Biobank participants (LV Mass)	N/A	1-SD	36 944 631
Ascending aorta distensibility	Pirruccello *et al*^29^	EUR	32 639	UK Biobank participants (Ascending aorta distensibility*)	N/A	1-SD	37 019 578
Myocardial native T1 time	Nauffal *et al*^30^	EUR	41 505	UK Biobank participants (Myocardial native T1 time*)	N/A	1-SD	37 081 215

* Not indexed to body surface area.

CH, clonal haematopoiesis; EUR, European; GWAS, Genome-Wide Association Study; ICD, International Classification of Diseases; LA Max, left atrial maximum volume; LATEF, left atrial total ejection fraction; LVEDV, left ventricular end-diastolic volume; LVEF, left ventricular ejection fraction; LV Mass, left ventricular mass; PMID, PubMed identifier; RA FAC, right atrial fractional area change; RA Max, right atrial maximum area; RVEDV, right ventricular end-diastolic volume; RVEF, right ventricular ejection fraction; VAF, variant allele fraction.

**Figure 1:**
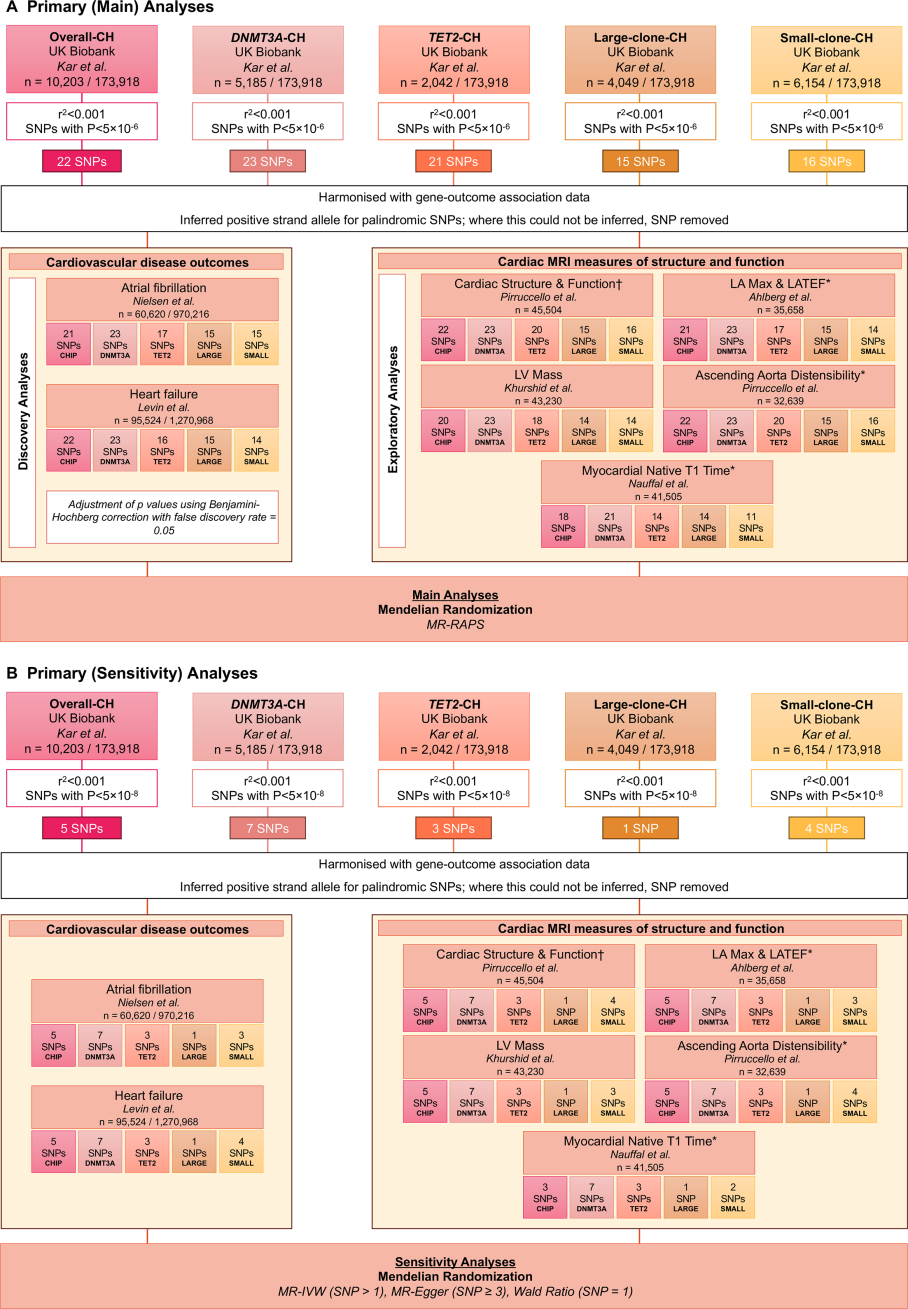
Primary analyses design using exposure instruments from Kar *et al.*^21^ CH, clonal haematopoiesis; LA Max, left atrial maximum volume; LATEF, left atrial total ejection fraction; LV Mass, left ventricular mass; MR-Egger, Mendelian randomisation using Egger regression; MR-IVW, Mendelian randomisation using the inverse variance weighted method; MR-RAPS, Mendelian Randomisation using a Robust Adjusted Profile Score; SNP, single nucleotide polymorphism. *, not indexed to body surface area; †, ascending aorta diameter, left ventricular end-diastolic volume (LVEDV), left ventricular ejection fraction (LVEF*), proximal pulmonary artery diameter, right atrial fractional area change (RA FAC*), right atrial maximum area (RA Max), right ventricular ejection fraction (RVEF), right ventricular end-diastolic volume (RVEDV).

For the primary analyses, gene-exposure association estimates were extracted from Kar *et al*’s^21^ GWAS with CH variants from the UK Biobank differentiated as (1) overall-CH, (2) *DNMT3A*-CH, (3) *TET2*-CH, (4) large-clone-CH and (5) small-clone-CH (figure 1). Gene-outcome association estimates of AF^24^ and HF^25^ were used for the discovery analysis, and 13 CMR phenotype estimates^26^,^30^ were used for the exploratory analysis.

After harmonisation, Mendelian Randomisation using a Robust Adjusted Profile Score^31^ (MR-RAPS) was used for primary analysis to estimate associations between genetically predicted CH and outcomes, which used relaxed selection criteria of p<5×10^−6^ and r^2^<0.001. Sensitivity analyses were carried out using Mendelian randomisation using the inverse variance weighted method^16^ (MR-IVW), Mendelian randomisation using Egger regression^32^ (MR-Egger), and, where appropriate, the Wald ratio method;^33^ these methods used conventional selection criteria of p<5×10^−8^ and r^2^<0.001.

The primary analysis was split into a discovery phase with CVD outcomes and an exploratory phase with CMR phenotypes. For CVD outcomes, results are presented as an OR with a respective 95% CI. All p values in the discovery analyses were Benjamini–Hochberg^34^ corrected for multiple testing with a 5% false discovery rate across all exposure-CVD outcome pairs. For CMR phenotypes, results are presented as a beta coefficient (β) with a 95% CI.

Replication analyses were performed using exposure instruments provided by Kessler *et al*^22^ from the UK Biobank defined as (1) overall-CH, (2) *DNMT3A*-CH and (3) *TET2*-CH (online supplemental figure 1). Furthermore, a validation analysis was conducted using exposure instruments of systolic blood pressure (SBP) from Evangelou *et al*^23^ to ensure consistency with recent observational evidence^35^ and to test the veracity of the approach (online supplemental figure 2). Finally, we conducted a phenome-wide scan of each single nucleotide polymorphism used as an instrumental variable in the analyses, to identify gene-exposure associations with alternative phenotypes.

The full methods for this study can be found in the online supplemental methods.

## Results

### CH and CVD risk (AF and HF)

**Figure 2:**
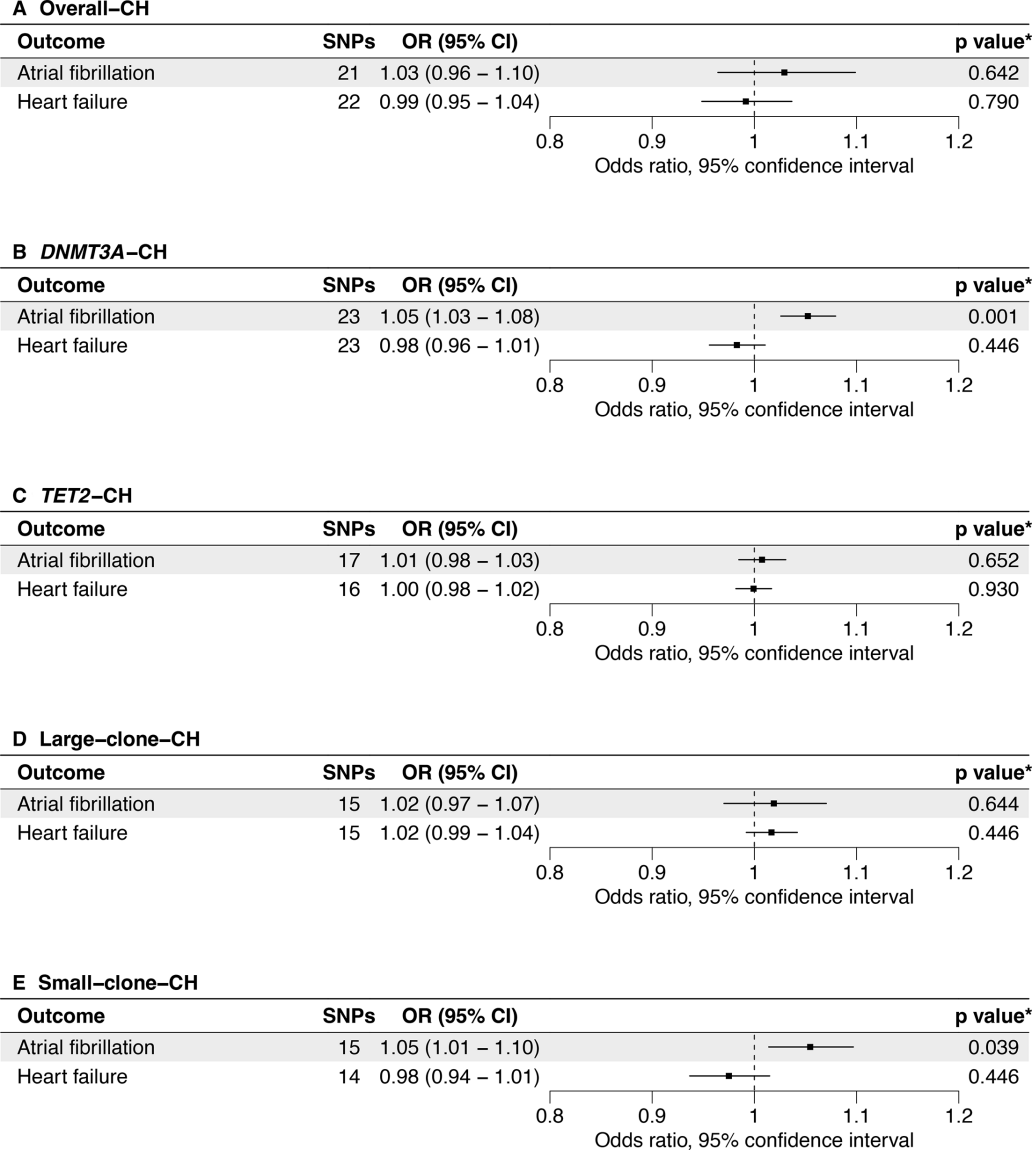
Primary analysis using MR-RAPS for the effects of CH from Kar *et al*^21^ on CVD outcomes. CH, clonal haematopoiesis; CVD, cardiovascular disease; MR-RAPS, Mendelian Randomisation using a Robust Adjusted Profile Score; SNP, single nucleotide polymorphism. *, adjusted values of p (false discovery rate = 5%).

The primary discovery analyses using Kar *et al*’s^21^ instruments (figure 2) showed that AF risk was increased by *DNMT3A*-CH (OR 1.05 (1.03 to 1.08), p=8.65×10^−4^) and small-clone-CH (OR 1.05 (1.01 to 1.10), p=3.91×10^−2^). Overall-CH, *TET2*-CH and large-clone-CH were also directionally associated with increased AF risk but were statistically non-significant. No significant associations were found with HF. Associations were consistent across the sensitivity analyses, with no evidence of directional pleiotropy (online supplemental table 2). Within the sensitivity analyses, we identified additional significant associations of overall-CH with increased AF risk (OR 1.09 (1.04 to 1.15), p=4.90×10^−4^), and *DNMT3A*-CH with decreased HF risk (OR 0.97 (0.95 to 1.00), p=2.86×10^−2^).

The replication analyses using Kessler *et al*’s^22^ instruments (online supplemental table 3) directionally supported the primary analyses. No statistically significant associations were identified with any CH subtype and CVD outcome. However, similar to the primary analyses, overall-CH, *DNMT3A*-CH and *TET2*-CH were directionally associated with increased AF risk. We identified no significant associations on replication sensitivity analyses and note no evidence of directional pleiotropy.

The validation analyses with increased SBP (by 5 mmHg) confirmed the suitability of our methods (online supplemental table 4) demonstrating significant associations with increased AF and HF risk (online supplemental figure 3).

### CH and CMR phenotypes

**Figure 3:**
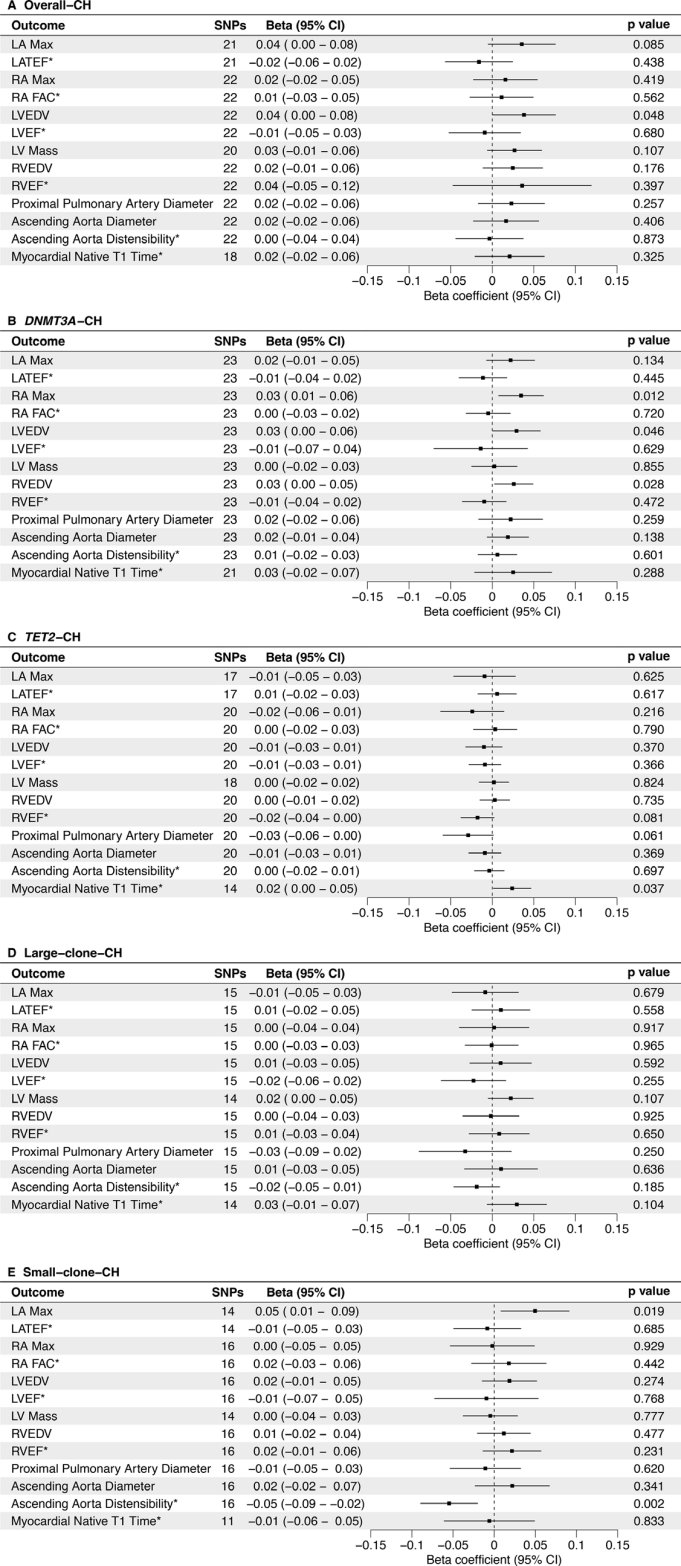
Primary analyses using MR-RAPS for effects of CH from Kar *et al*^21^ on CMR phenotypes. Beta, beta coefficient; CH, clonal haematopoiesis; CMR, cardiovascular magnetic resonance; LA Max, left atrial maximum volume; LATEF, left atrial total ejection fraction; LV Mass, left ventricular mass; LVEDV, left ventricular end-diastolic volume; LVEF, left ventricular ejection fraction; MR-RAPS, Mendelian Randomisation using a Robust Adjusted Profile Score; RA FAC, right atrial fractional area change; RA Max, right atrial maximum area; RVEDV, right ventricular end-diastolic volume; RVEF, right ventricular ejection fraction; SNP, single nucleotide polymorphism. *not indexed to body surface area.

The exploratory analyses with Kar *et al*’s^21^ instruments and CMR phenotypes are shown in figure 3. Overall-CH was associated with increased left ventricular end-diastolic volume (LVEDV) (*β* 0.04 (0.00 to 0.08), p=4.81×10^−2^). *DNMT3A*-CH was associated with increased right atrial maximum area (RA Max) (*β* 0.03 (0.01 to 0.06), p=1.16×10^−2^), LVEDV (*β* 0.03 (0.00 to 0.06), p=4.57×10^−2^) and right ventricular end-diastolic volume (RVEDV) (*β* 0.03 (0.00 to 0.05), p=2.79×10^−2^). *TET2*-CH was associated with increased myocardial native T1 time (*β* 0.02 (0.00 to 0.05), p=3.71×10^−2^). Small-clone-CH was associated with increased left atrial maximum volume (LA Max) (*β* 0.05 (0.01 to 0.09), p=1.89×10^−2^) and decreased ascending aorta distensibility (*β* −0.05 (−0.09 to −0.02), p=1.88×10^−3^). No significant associations with large-clone-CH were identified. Associations were consistent on sensitivity analyses, with no evidence of directional pleiotropy (online supplemental table 5). Within the sensitivity analyses, we identified additional significant associations; overall-CH was associated with increased RVEDV (*β* 0.06 (0.02 to 0.11), p=5.48×10^−3^) and proximal pulmonary artery diameter (*β* 0.07 (0.02 to 0.12), p=8.72×10^−3^). *TET2*-CH was associated with increased LVEDV (*β* 0.03 (0.00 to 0.07), p=4.58×10^−2^) and proximal pulmonary artery diameter (*β* 0.05 (0.02 to 0.09), p=3.78×10^−3^). Finally, small-clone-CH was associated with increased LVEDV (*β* 0.06 (0.01 to 0.10), p=1.36×10^−2^).

The replication analyses, using Kessler *et al*’s^22^ instruments, were directionally supportive of the primary exploratory analyses. These were also consistent on sensitivity analyses, and we note no evidence of directional pleiotropy (online supplemental table 6). Furthermore, the sensitivity analyses identified an association of *DNMT3A*-CH with increased RA Max (*β* 0.05 (0.01 to 0.09), p=1.80×10^−2^), RVEDV (*β* 0.04 (0.01 to 0.07), p=1.60×10^−2^) and ascending aorta diameter (*β* 0.03 (0.00 to 0.05), p=3.14×10^−2^). Finally, *TET2*-CH was associated with increased LVEDV (*β* 0.04 (0.01 to 0.07), p=8.04×10^−3^), LV Mass (*β* 0.04 (0.01 to 0.07), p=2.92×10^−3^) and RVEDV (*β* 0.04 (0.01 to 0.07), p=6.77×10^−3^).

The validation analyses with increased SBP (by 5 mmHg) confirmed method suitability (online supplemental table 7) demonstrating significant associations with increased LA Max, LVEDV, LVEF, LV Mass, RVEF and ascending aorta diameter, and associations with decreased LATEF, ascending aorta distensibility and myocardial native T1 time (online supplemental figure 4).

### Phenome-wide analyses

The results of the phenome-wide analyses are presented in online supplemental table 8.

## Discussion

CH has been widely associated with heightened CVD risk in observational analyses,^5^,^11^ supported by experimental models of *DNMT3A*^36 37^ and *TET2*^537^,^40^ mediated CH. Although extremely useful for mechanistic insight, such models often reflect rare and more extreme CH phenotypes, with effects on CVD outcomes diminished, or even lost, with heterozygotic mutations, making extrapolation difficult to human populations with a greater heterozygotic burden.^41^ Also, the close relationship between ageing and both CH and CVD makes it difficult to eliminate residual confounding in observational studies, particularly as chronological age adjustment cannot fully account for impacts of biological age.^14^ Therefore, causal links between CH and CVD remain uncertain.

Using Mendelian randomisation methods to mitigate residual confounding and reverse causation, our study triangulates previous observational results and provides new evidence supporting potential causal associations of CH with AF. We further extend existing evidence, using CMR-derived phenotypes to characterise, for the first time, adverse cardiovascular remodelling patterns associated with CH; our findings indicate genetic associations with larger atrial and ventricular sizes, higher LV mass, higher myocardial native T1 time, and lower aortic distensibility, although in a subtype specific manner. The robustness of our methods and findings is demonstrated across multiple validation and replication analyses.

Amongst subtypes examined, *DNMT3A*-CH exhibited the largest and most consistent associations with AF risk and adverse cardiovascular remodelling. This may indicate heterogenic associations with CVD across CH subtypes, with dominance of individual driver genes conferring differing downstream consequences. This is supported by weaker, less consistent associations for overall-CH, a composite category of mutants, despite greater statistical power. However, this is unsurprising given driver genes have differing functional roles, with sometimes biologically opposing functions. For example, the key function of *DNMT3A* is DNA methylation leading to transcription repression,^42^ whereas *TET2* is an important epigenetic regulator, causing DNA demethylation and widespread gene activation.^43^ Despite this, studies have found a paradoxical convergence of *DNMT3A* and *TET2*-mediated effects.^5 36^ Although we did not find significant associations between *TET2*-CH and AF, this contradicts previous observational studies;^39 44^ Ahn *et al*^44^ demonstrated heightened AF risk in individuals with *TET2*-CH and large-clone-CH. Given that *TET2*-CH is associated with increased AF risk across sensitivity analyses and that there are fewer individuals within this subtype, lack of associations within the primary analyses is very likely to be due to limited power.

Atrial remodelling is integral in arrhythmia predisposition and AF pathogenesis—which frames the key finding of *DNMT3A*-CH and small-clone-CH being concomitantly associated with AF and larger atrial size. Given higher proportions of *DNMT3A* mutants in small-clone-CH versus large-clone-CH subtypes within the primary analysis^21^ instruments (53% *DNMT3A*, 20% *TET2* mutants in small clones; 46% *DNMT3A*; 21% *TET2* mutants in large clones), these similar findings are expected and support previous findings of increased atrial size^44^ and AF incidence for CH subtypes.^6^ Increased atrial size robustly associates with heightened AF risk^45 46^ and is an established precursor to AF onset,^47^ with dilatation and fibrosis associated with conduction irregularity. However, AF itself can lead to atrial dilatation^48^ which further begets AF in a vicious spiral. Mechanistically, CH may predispose individuals to atrial dilatation and myopathy which may occur alongside other risk factors. In an angiotensin-II hypertension murine model,^37^ *DNMT3A* and *TET2* mutations induced structural and functional changes similar to those described. Inflammatory cytokines are increasingly recognised as integral in driving cardiac remodelling and AF risk. Increased high-sensitivity C reactive protein (hs-CRP) has been identified in CH,^49^ and associations with AF are attenuated on hs-CRP^50^ adjustment, suggesting a potential mediating role. Furthermore, Nlrp3 inflammasome activation has been proposed to promote arrhythmogenesis through altered cardiomyocyte calcium handling in mice with *TET2* mutations.^38^ Notably, in the current study, *TET2*-CH was associated with an increase in myocardial native T1 time, a proxy of myocardial fibrosis associated with CVD risk,^51^ and has been described in observational studies of UK Biobank participants.^52^ Although not yet studied in *DNMT3A*-CH experimental models, Nlrp3 inflammasome activating mutations have been found to have convergent effects to *TET2* mutations in macrophages,^36^ suggesting a similar phenomenon may occur. We highlight this as an area for further investigation.

HF has been implicated as a consequence of CH in observational studies and experimental models^7^,^1053^ which is suggested to be driven by similar inflammatory mechanisms to those previously discussed. Generally, our analyses did not find significant associations between any CH subtype and HF, which is supported by some previous observational studies^7^,^10^ and MR analyses.^21^ In one sensitivity analysis, we found a lower rate of HF with *DNMT3A*-CH, which may be explained by survival bias among the outcome GWAS^25^ given that previous observational analyses have demonstrated increased mortality in *DNMT3A*-CH-driven HF.^7^ *DNMT3A*-CH did, however, associate with increased LVEDV and RVEDV, with some suggestion of similar volumetric changes and increased myocardial T1 time for *TET2-*CH. These structural changes may represent early compensatory changes in preclinical HF,^54 55^ and the benefits of lower imaging threshold in these individuals warrant investigation.

The strength of our study arises from leveraging large-scale GWAS summary statistics for exposures and outcomes. Furthermore, we use MR-RAPS,^31^ which can increase statistical power by including conventionally ‘weaker instruments’ and overcome current challenges in CH investigations. Similar CH investigations^56^ have used this approach and, with consideration of MR-RAPS limitations, can provide evidence supporting causality.^31^ Using such genetic inferential techniques helps to support and validate current literature which has described observational associations between CH subtypes and adverse remodelling.^52^ Finally, the validation analyses support our approach with associations consistent with current knowledge of SBP as a risk factor for AF^57 58^ and HF,^59^ alongside recent observational evidence which corroborates findings of adverse CMR phenotypes.^35^

### Limitations

While helpful in removing systematic biases, MR cannot completely exclude the potential for horizontal and/or vertical pleiotropy,^60^ although appreciation of relevant potential biological mechanisms can aid in understanding these limitations. An obvious limitation is that telomere-mediated biological ageing can be argued to influence CH and outcome associations. Telomere lengthening^61^ has been associated with CH subtypes, whereas shortening is associated with CVD.^62^ However, to cause significant pleiotropy, the CH and CVD relationships with telomere length would need to be directionally consistent. Moreover, the lack of significant MR-Egger intercepts suggests significant pleiotropy is unlikely,^32^ although our findings should be interpreted with this in mind. The accuracy of our results further rests on the definition within the original publications^21 22^ and recognise this remains controversial.^63^

We note that significant associations were not replicated in the study by Kessler *et al*,^22^ which could result from regression dilution. Additionally, we acknowledge that results from the study by Kar *et al*^21^ may be interpreted as ‘random positive’, although we argue this is unlikely given biological and directional consistency of results. Associations with *TET2* were limited, and we acknowledge that current literature with observational studies^64^ has identified *TET2*-CH to be associated strongly with HF. Insufficient power is the most likely explanation, given that subtypes with significant associations had higher instrument strength. A repeated analysis leveraging targeted CH sequencing is likely to yield more insights, as our ability to detect the impact of smaller clones is limited with whole-exome sequencing;^63^ we are unable to do this at present given the lack of publicly available summary statistics of such data, and highlight this as an area for further investigation. In addition, genetics-based inferential studies are vulnerable to instrumental variable assumption violations, which we mitigate by careful instrument selection and by using a variety of MR methods, but we note this cannot be fully eliminated. For example, to address the first instrumental variable assumption (that variants must be able to predict the exposure), we calculate F-statistics to identify weak instruments. We did not find any variants with an F-statistic <10, providing no evidence of weak instruments. Furthermore, in order to reduce violations of the second instrumental variable assumption (no common causes of the genetic variant and the outcome), this analysis is limited to individuals of European ancestry to reduce confounding from population stratification. However, this does mean that validation in other groups is needed, given that we are unable to extrapolate our findings to populations of other ancestries. Finally, although we use a single data set for exposures and outcomes (the UK Biobank), recent evidence suggests risk of biased estimates due to correlation is minimal when using two-sample MR methods in large data sets.^65^

## Conclusions

This study supports potential causal associations between *DNMT3A*-CH and small-clone-CH with AF risk and atrial dilatation. Furthermore, we identify, for the first time, associations of *DNMT3A*-CH with left and right ventricular dilatation and of *TET2-*CH with increased myocardial native T1 time which may represent manifestations of preclinical HF. These structural changes may indicate a potential window of opportunity for intervention, such as by risk stratification and early preventative strategies to improve patient outcomes. Our findings extend the growing literature regarding the cardiovascular sequelae of CH and structural abnormalities. Further research is required to establish the pathophysiological mechanisms underlying increased CVD risk and their clinical implications, and the best interventions to attenuate such risk.

## Supplementary material

10.1136/openhrt-2025-003602 online supplemental file 1

10.1136/openhrt-2025-003602 online supplemental table 1

## Data Availability

Data are available in a public, open access repository.
