# Supplementary material for: Causal relevance of clonal haematopoiesis with cardiac disease and adverse remodelling: a Mendelian randomisation study
Source: Open Heart. 2025 Oct 17;12(2):e003602. doi: 10.1136/openhrt-2025-003602 (PMC12542733; doi:10.1136/openhrt-2025-003602)
Supplement: online supplemental file 1 [file openhrt-12-2-s001.docx]

**Supplement**

[**Supplemental Methods** 2](#_Toc209466091)

[**Supplemental Figure 1** – Replication analyses design using exposure instruments from Kessler et al. 7](#_Toc209466092)

[**Supplemental Figure 2 –** Validation analyses design using exposure instruments from Evangelou et al. 9](#_Toc209466093)

[**Supplemental Figure 3 –** Validation analyses using MR-RAPS for effects of SBP from Evangelou et al. on CVD outcomes. 11](#_Toc209466094)

[**Supplemental Figure 4 –** Validation analyses using MR-RAPS for effects of SBP from Evangelou et al. on CMR phenotypes 12](#_Toc209466095)

[**References** 13](#_Toc209466096)

# Supplemental Methods

## Ethics and Data Access

This study used publicly available Genome-Wide Association Study (GWAS) summary data available to download at cited sources. Ethical approval and participant consent were obtained in the original publications. It was not appropriate or possible to involve patients or the public in the design, or conduct, or reporting, or dissemination plans of our research. A summary of sources is provided in Table 1.

This study is reported according to the Strengthening the Reporting of Observational Studies in Epidemiology using Mendelian Randomization (STROBE-MR) Guidelines^1^. All statistical analyses were performed using R version 4.3.1^2^ using the TwoSampleMR^3^, MendelianRandomization^4^ and mr.raps packages.

## Study Exposures

For the primary analyses, instruments were extracted from Kar et al.’s^5^ GWAS with clonal haematopoiesis (CH) variants from the UK Biobank differentiated as (1) overall-CH, (2) *DNMT3A*-CH, (3) *TET2*-CH, (4) large-clone-CH, and (5) small-clone-CH (Figure 1).

Replication analyses were performed utilising instruments provided by Kessler et al.’s^6^ GWAS from the UK Biobank differentiated as (1) overall-CH, (2) *DNMT3A*-CH and (3) *TET2*-CH (Supplemental Figure 1).

Instrument strength was quantified using F-statistics (**Supplemental Table 1**) using the formula:

$$F=\frac{(n-k-1)}{k} \frac{(R^{2})}{(1-R^{2})}$$

where $R^{2}$ is the explained variance in the regression of all single nucleotide polymorphisms (SNPs), *n* is the number of participants in the study, *k* is the number of instrumental variants. The $R^{2}$ was calculated as the sum of SNP-wise $R^{2}$ of instruments, which is obtained as follows:

$R^{2}= \frac{F}{(n-2+F)}$ with $F=\left( \frac{\beta}{SE(\beta)} \right)^{2}$

where *β* represents the effect size of the genetic variant in the exposure GWAS, and *SE*(*β* ) represents the standard error of the effect size of the genetic variant in the exposure GWAS.

## Study Outcomes

Genetic association estimates for clinical cardiovascular disease (CVD) outcomes were extracted from the largest available GWAS summary statistics for atrial fibrillation (AF, 60,620 cases and 970,216 controls)^7^ and heart failure (HF, 95,524 cases and 1,270,968 controls)^8^ in populations of European ancestry (Table 1).

We included established cardiovascular magnetic resonance (CMR) phenotypes representing structure and function of all four cardiac chambers, myocardial tissue character, and central vasculature. Genetic association estimates for CMR phenotypes were extracted from the largest available GWAS summary statistics, which were all based on the UK Biobank cohort (Table 1). The majority were derived from Pirruccello et al.’s^9^ study of 45,504 individuals which included: right atrial maximum area (RA Max), right atrial fractional area change (RA FAC), left ventricular end-diastolic volume (LVEDV), left ventricular ejection fraction (LVEF), right ventricular end-diastolic volume (RVEDV), right ventricular ejection fraction (RVEF), proximal pulmonary artery diameter and ascending aorta diameter. Estimates for left atrial maximum volume (LA Max) and left atrial total ejection fraction (LATEF) were derived from Ahlberg et al.’s^10^ study of UK Biobank participants including 35,658 individuals. Estimates for left ventricular mass (LV Mass) were derived from Khurshid et al.’s^11^ study of 43,230 individuals. Estimates for ascending aorta distensibility were extracted from Pirruccello et al.’s^12^ study of 32,639 individuals. Finally, estimates for myocardial native T1 time were extracted from Nauffal et al.’s^13^ study of 41,505 individuals. In the GWAS summary statistics, all outcomes were indexed by body surface area; except RA FAC, LVEF, RVEF and LATEF which are dimensionless, ascending aorta distensibility which is measured in ×10^-3^ mmHg^-1^, and myocardial native T1 time which is measured in milliseconds.

## Statistical Analyses

Gene-exposure association data was individually harmonised with gene-outcome association data using the TwoSampleMR^3^ package in R^2^. During harmonisation, the positive strand allele was inferred where possible, and if not, the single nucleotide polymorphism (SNP) was dropped from further analysis.

Mendelian Randomization using a Robust Adjusted Profile Score^14^ (MR-RAPS) was used for the primary analyses to estimate associations between genetically-predicted CH and outcomes. This facilitated increased statistical power by including conventionally ‘weaker’ instruments by relaxing selection criteria to *P* < 5×10^−6^ and r^2^ < 0.001. The primary analysis was split into a discovery phase, with CVD outcomes, and an exploratory phase, with CMR phenotypes. For CVD outcomes, results are presented as an odds ratio (OR) with a respective 95% confidence interval (CI). All p values in the discovery analyses were Benjamini-Hochberg^15^ corrected for multiple testing with a 5% false discovery rate across all exposure-CVD outcome pairs. For CMR phenotypes, results are presented as a Beta coefficient (β) and 95% CIs.

Sensitivity analyses were carried out using Mendelian Randomization using the Inverse Variance Weighted method^16^ (MR-IVW), where SNPs > 1, as well as Mendelian Randomization using Egger regression^17^ (MR-Egger), where SNPs ≥ 3. In the case of a single SNP being available for analysis, the Wald Ratio^18^ method was used. These methods used conventional selection criteria of *P* < 5×10^−8^ and r^2^ < 0.001.

## Assumptions

A core assumption of the MR-RAPS approach is the absence of directional pleiotropy in genetic instruments. Horizontal pleiotropy, where genetic variants act through additional pathways, can still provide valid results if balanced throughout variables^14^. Sensitivity analyses, using MR-IVW and MR-Egger were performed, where possible, to investigate this.

The core assumptions of MR-IVW^16^ include: relevance, independence and exclusion restriction. We address the first assumption, that the variants must be able to predict the exposure by calculating F-statistics, with values <10 signifying the presence of a weak instrument (Supplemental Table 1). The second assumption, that there must be no common causes of the genetic variant and the outcome, is unable to be formally tested; however, we restrict our analyses to individuals of European ancestry, to reduce the impact of confounding from population stratification. Finally, the third assumption, that variants should only influence the outcome through the exposure, and not directly or through alternative phenotypes, was addressed by including MR-Egger as a sensitivity analysis, to establish consistency with the primary analyses. Furthermore, the MR-Egger method can be used to identify the presence of directional pleiotropy under a weaker assumption that the instrument strength is independent of direct effects (InSIDE assumption)^17^. A significant p value on the MR-Egger intercept suggests the presence of directional pleiotropy.

## Replication and Validation Analyses

All analyses were repeated on exposures from Kessler et al.^6^ as part of replication analyses. We selected Kar et al.^5^ for the purpose of primary analysis given the use of a large sample size with whole-exome sequencing data, followed by a stringent approach to significance threshold and subtype-specific analyses. In contrast, Kessler et al.^6^, despite using a larger sample size, focused on both common and rare variants which increased the complexity of data handling. Although this provided the added advantage of the ability to uncover rare variant associations, it made further MR analyses arguably less robust and more challenging to interpret given the core assumptions that must be met to infer causality. Furthermore, validation analyses for our methodology (**Supplemental Figure 2**) were conducted using systolic blood pressure (SBP) from Evangelou et al.^19^, to ensure consistency with recent observational evidence^20^ and to test the veracity of our approach.

## Phenome Wide Analyses

We conducted a phenome-wide scan of SNPs used as instrumental variables in our analyses, to identify gene-exposure associations with alternative phenotypes, using the NHGRI-EBI GWAS Catalog^21^ with a significance threshold of *P* < 5×10^−8^.

# Supplemental Figure 1 – Replication analyses design using exposure instruments from Kessler et al.

CH = clonal haematopoiesis, CMR = cardiovascular magnetic resonance, CVD = cardiovascular disease, LA Max = left atrial maximum volume, LATEF* = left atrial total ejection fraction, LV Mass = left ventricular mass, MR-Egger = Mendelian Randomization using Egger regression, MR-IVW = Mendelian Randomization using the Inverse Variance Weighted method, MR-RAPS = Mendelian Randomization using a Robust Adjusted Profile Score, SNP = single nucleotide polymorphism. †ascending aorta diameter, left ventricular end-diastolic volume (LVEDV), left ventricular ejection fraction (LVEF*), proximal pulmonary artery diameter, right atrial fractional area change (RA FAC*), right atrial maximum area (RA Max), right ventricular ejection fraction (RVEF), right ventricular end-diastolic volume (RVEDV). *not indexed to body surface area.

# Supplemental Figure 2 – Validation analyses design using exposure instruments from Evangelou et al.

CMR = cardiovascular magnetic resonance, CVD = cardiovascular disease, LA Max = left atrial maximum volume, LATEF* = left atrial total ejection fraction, LV Mass = left ventricular mass, MR-Egger = Mendelian Randomization using Egger regression, MR-IVW = Mendelian Randomization using the Inverse Variance Weighted method, MR-RAPS = Mendelian Randomization using a Robust Adjusted Profile Score, SNP = single nucleotide polymorphism. †ascending aorta diameter, left ventricular end-diastolic volume (LVEDV), left ventricular ejection fraction (LVEF*), proximal pulmonary artery diameter, right atrial fractional area change (RA FAC*), right atrial maximum area (RA Max), right ventricular ejection fraction (RVEF), right ventricular end-diastolic volume (RVEDV). *not indexed to body surface area.

# Supplemental Figure 3 – Validation analyses using MR-RAPS for effects of SBP from Evangelou et al. on CVD outcomes.

CI = confidence interval, CVD = cardiovascular disease, MR-RAPS = Mendelian Randomization using a Robust Adjusted Profile Score, OR = odds ratio, SBP = systolic blood pressure, SNP = single nucleotide polymorphism.

# Supplemental Figure 4 – Validation analyses using MR-RAPS for effects of SBP from Evangelou et al. on CMR phenotypes

Beta = beta coefficient, CI = confidence interval, CMR = cardiovascular magnetic resonance, LA Max = left atrial maximum volume, LATEF* = left atrial total ejection fraction, LV Mass = left ventricular mass, LVEDV = left ventricular end-diastolic volume, LVEF* = left ventricular ejection fraction, MR-RAPS = Mendelian Randomization using a Robust Adjusted Profile Score, RA FAC* = right atrial fractional area change, RA Max = right atrial maximum area, RVEDV = right ventricular end-diastolic volume, RVEF* = right ventricular ejection fraction, SBP = systolic blood pressure, SNP = single nucleotide polymorphism. *not indexed to body surface area.

# References

1. Skrivankova VW, Richmond RC, Woolf BAR, et al. Strengthening the reporting of observational studies in epidemiology using mendelian randomisation (STROBE-MR): explanation and elaboration. *BMJ* 2021;375:n2233. doi: 10.1136/bmj.n2233 [published Online First: 20211026]

2. R: A Language and Environment for Statistical Computing [program]: R Foundation, 2023.

3. Hemani G, Zheng J, Elsworth B, et al. The MR-Base platform supports systematic causal inference across the human phenome. *Elife* 2018;7 doi: 10.7554/eLife.34408 [published Online First: 20180530]

4. Yavorska OO, Burgess S. MendelianRandomization: an R package for performing Mendelian randomization analyses using summarized data. *Int J Epidemiol* 2017;46(6):1734-39. doi: 10.1093/ije/dyx034

5. Kar SP, Quiros PM, Gu M, et al. Genome-wide analyses of 200,453 individuals yield new insights into the causes and consequences of clonal hematopoiesis. *Nat Genet* 2022;54(8):1155-66. doi: 10.1038/s41588-022-01121-z

6. Kessler MD, Damask A, O’Keeffe S, et al. Common and rare variant associations with clonal haematopoiesis phenotypes. *Nature* 2022;612(7939):301-09. doi: 10.1038/s41586-022-05448-9

7. Nielsen JB, Thorolfsdottir RB, Fritsche LG, et al. Biobank-driven genomic discovery yields new insight into atrial fibrillation biology. *Nat Genet* 2018;50(9):1234-39. doi: 10.1038/s41588-018-0171-3

8. Levin MG, Tsao NL, Singhal P, et al. Genome-wide association and multi-trait analyses characterize the common genetic architecture of heart failure. *Nat Commun* 2022;13(1):6914. doi: 10.1038/s41467-022-34216-6

9. Pirruccello JP, Di Achille P, Nauffal V, et al. Genetic analysis of right heart structure and function in 40,000 people. *Nat Genet* 2022;54(6):792-803. doi: 10.1038/s41588-022-01090-3

10. Ahlberg G, Andreasen L, Ghouse J, et al. Genome-wide association study identifies 18 novel loci associated with left atrial volume and function. *Eur Heart J* 2021;42(44):4523-34. doi: 10.1093/eurheartj/ehab466

11. Khurshid S, Lazarte J, Pirruccello JP, et al. Clinical and genetic associations of deep learning-derived cardiac magnetic resonance-based left ventricular mass. *Nat Commun* 2023;14(1):1558. doi: 10.1038/s41467-023-37173-w

12. Pirruccello JP, Rämö JT, Choi SH, et al. The Genetic Determinants of Aortic Distention. *J Am Coll Cardiol* 2023;81(14):1320-35. doi: 10.1016/j.jacc.2023.01.044

13. Nauffal V, Di Achille P, Klarqvist MDR, et al. Genetics of myocardial interstitial fibrosis in the human heart and association with disease. *Nat Genet* 2023;55(5):777-86. doi: 10.1038/s41588-023-01371-5 [published Online First: 20230420]

14. Zhao Q, Wang J, Hemani G, et al. Statistical inference in two-sample summary-data Mendelian randomization using robust adjusted profile score. *Ann Statist* 2020;48(3):1742-69. doi: 10.1214/19-AOS1866

15. Benjamini Y, Hochberg Y. Controlling the False Discovery Rate: A Practical and Powerful Approach to Multiple Testing. *J R Stat Soc Series B Stat Methodol* 1995;57(1):289-300. doi: 10.1111/j.2517-6161.1995.tb02031.x

16. Burgess S, Butterworth A, Thompson SG. Mendelian Randomization Analysis With Multiple Genetic Variants Using Summarized Data. *Genet Epidemiol* 2013;37(7):658-65. doi: 10.1002/gepi.21758

17. Burgess S, Thompson SG. Interpreting findings from Mendelian randomization using the MR-Egger method. *Eur J Epidemiol* 2017;32(5):377-89. doi: 10.1007/s10654-017-0255-x

18. Wald A. The Fitting of Straight Lines if Both Variables are Subject to Error. *Ann Math Statist* 1940;11(3):284-300. doi: 10.1214/aoms/1177731868

19. Evangelou E, Warren HR, Mosen-Ansorena D, et al. Genetic analysis of over 1 million people identifies 535 new loci associated with blood pressure traits. *Nat Genet* 2018;50(10):1412-25. doi: 10.1038/s41588-018-0205-x

20. Elghazaly H, McCracken C, Szabo L, et al. Characterizing the hypertensive cardiovascular phenotype in the UK Biobank. *Eur Heart J Cardiovasc Imaging* 2023;24(10):1352-60. doi: 10.1093/ehjci/jead123

21. Sollis E, Mosaku A, Abid A, et al. The NHGRI-EBI GWAS Catalog: knowledgebase and deposition resource. *Nucleic Acids Res* 2023;51(D1):D977-d85. doi: 10.1093/nar/gkac1010
